# Influence of language use on migrant workers’ willingness to Urban settlement-based on the CLDS 2016 survey data

**DOI:** 10.1371/journal.pone.0294906

**Published:** 2023-11-29

**Authors:** Longjunjiang Huang, Xian Liang, Lishan Li, Hui Xiao, Fangting Xie

**Affiliations:** 1 School of Business Administration, Zhongnan University of Economics and Law, Wuhan, China; 2 School of Economics and Management, Beijing Forestry University, Beijing, China; 3 School of Economics and Management, Jiangxi Agricultural University, Jiangxi, China; 4 School of Economics and Management, Zhejiang A & F University, Hangzhou, China; 5 Research Academy for Rural Revitalization of Zhejiang Province, Zhejiang A & F University, Hangzhou, China; Noida International University, INDIA

## Abstract

In the framework of China new urbanization construction, migrant workers are of great significance for urban sustainable development. Based on the data of CLDS2016, we used the multi- -ordered Logistics model to effectively explore the influence of migrant workers ’ language use on the urban settlement will. The research results showed that the mastery of the urban dialect and the language used after work had a significant impact on the willingness of the migrant workers in the urban settlement, while the language used at work had no significant impact on the migrant workers’ willingness to settle in cities. Therefore, policy implications are proposed on improving the social integration of migrant workers, enhancing the sense of belonging, and removing the obstacles and doubts in the process of urban settlement.

## 1. Introduction

According to the 2019 Migrant Worker Monitoring Survey Report released by the National Bureau of Statistics in March 2020, the total number of migrant workers reached about 290 million in 2019, including 116.52 million local migrant workers and 174.25 million migrant workers out of the country. Among the migrant workers working abroad nationwide, the proportion of migrant workers employed in the province to the proportion of migrant workers going abroad has increased, and migrant workers moving to the city have a higher sense of belonging to the city where they are located and participate more actively in various activity organizations [1]. In the context of rural revitalization, the State Council issued the "Strategic Plan for Rural Revitalization (2018–2022)", in which it is clearly stated that "we should promote the orderly citizenship of the agricultural transfer population who can live and work stably in cities and towns". As a major component of urban construction, migrant workers have made great contributions to urban construction, but they are constantly marginalized in terms of their sense of belonging to the city, which has become an obstacle to the transformation and development of urbanization in China [2]. The settlement of migrant workers in cities is of great significance in fundamentally solving the "three rural problems", promoting the healthy development of urbanization, and expanding domestic demand [3]. The migration of a large number of young and strong rural laborers to work in cities keeps the demographic dividend in cities, and the process of their citizenship is to fundamentally improve the allocation of resources, which is conducive to further coordination of urban and rural development. The continuous influx of young and strong rural laborers to cities and towns, and the inevitable choice of cities and towns to absorb the employment of migrant workers and promote their transformation into citizens to accelerate the urbanization process. Migrant workers are the solid force of urban industrial development, and whether they can realize citizenship is related to the stability of migrant workers and the development of urbanization.

The complexity of China’s long-term urban-rural dual system affects migrant workers’ willingness to settle. It has been shown that migrant workers’ willingness to settle in cities is influenced by multiple factors [4,5] and is the result of a combination of subjective and objective measures [6]. In addition, the sense of social integration in urban life is also an important influencing factor in their willingness to settle. A review of the literature reveals that the language use of migrant workers affects their income [7], social identity [8], and sense of urban integration [9]. However, fewer scholars have conducted empirical investigations on the language use and settlement intentions of migrant workers. The current literature on language use and urban integration among migrant workers is mostly limited to descriptive analysis and correlational analysis, without any in-depth exploration of the intrinsic influence mechanisms. In terms of language use of migrant workers, most of them use indicators such as proficiency in Mandarin, proficiency in their home language, and familiarity with urban dialects [10,11], without further delineating the occasions and times of language use, such as whether the difference between the language mainly used by migrant workers at work and the language mainly used for after-work communication has a significant effect on their urban settlement intentions. Therefore, as a supplement to the existing studies, the innovation of this paper is that it distinguishes and compares the languages mainly used by migrant workers at work and after work, and explores the paths of different language use habits on migrant workers’ intention to settle in cities in these two contexts respectively.

## 2. Theoretical analysis and research hypothesis

Since the reform and opening up, the rapid economic development of China’s eastern coastal cities has attracted a large-scale population movement, and the scale of the urban-rural population movement has expanded, with a large amount of surplus agricultural labor flowing into cities. However, the urban-rural dual structure policy implemented in China for a long time has restricted the migration choice of rural migrants, and Zhu Y (2007) pointed out that the low level of social security and the high-risk expectation of the future has led to the circular mobility of migrant workers [12]. Circular mobility refers to the fact that throughout the migration process, the migrant population maintains close ties with the family in the place of migration [13]. With the development of time and the economy, migrant workers were more in circular mobility in the early days, but more and more migrant workers are now seeking the opportunity to settle permanently in cities. The willingness of migrant workers to settle in cities is a comprehensive consideration of their conditions [14].

The factors affecting migrant workers’ willingness to settle in cities are complex and multiple, and this paper mainly reviews them from two aspects: internal and external factors. From external factors, macro policies such as household registration system [15], land policy [16], and fiscal policy [17], all affect migrant workers’ willingness to settle in cities to some extent. The macroscopic dual household registration restricts the citizenship process of migrant workers [18], while the improvement of the residence permit management system has created better conditions for urban farmers to enjoy urban social benefits. The current land policy restricts the citizenship of migrant workers, while rural land transfer will promote the citizenship of migrant workers. Improving the current fiscal system can better alleviate the dilemma of agricultural migration citizenship [17]. Meanwhile, the convenience of urban public services can significantly promote migrant workers’ willingness to settle in cities [19]. The solution to the education problem of migrant workers’ children is conducive to promoting migrant workers’ settlement decisions [20]. In terms of internal factors, personal characteristics, family characteristics, and human capital of migrant workers all have an impact on migrant workers’ willingness to settle in cities. Among the personal characteristics of migrant workers, age [21], gender [22], and marital status [23] were shown to have an impact on their settlement intentions. Among family characteristics, family care [24], family size [25], and children’s education all have an impact on settlement intentions. Among the human capital characteristics of migrant workers [20], the factors of educational attainment [26], duration of working in the city [27], and the presence of vocational training experience [7] are usually considered. It is worth noting that a stable and promising job implies income and livelihood stability, and the occupational stability of migrant workers also contributes to their willingness to settle [12].

The language use of migrant workers is a social phenomenon that can be directly observed in this group, and several scholars have analyzed the impact of the language use of migrant workers on their income, social identity, and urban integration [11]. After investigating the language life and urban integration of migrant workers in Shanghai, Yu Weiqi et al. found that the improvement of migrant workers’ Mandarin and urban dialect proficiency helped to reduce their psychosocial distance from urban residents and enhance their identity in the city; among them, proficiency in Shanghai dialect had a more significant effect on reducing psychosocial distance and enhancing identity [28]. Regarding the association between Mandarin use and social identity, some scholars have also put forward different views, arguing that there is no necessary connection between migrant workers’ Mandarin use in the city and their social identity. For example, Fu Yirong et al. used 228 survey data of migrant workers in Shanghai to show that the new generation and the older generation of migrant workers generally speak Mandarin in the city and speak local dialect when they return to their hometown, and such language use habits do not have much relationship with their social identity to the city and the countryside [28].

Domestic sociolinguists have made a series of observations to explore the language use of migrant workers in the city and found the following phenomena. New and old migrant workers commonly use Mandarin in their work and social activities, but the older generation of migrant workers are influenced by their long-standing language habits and are susceptible to the influence of their previously formed language habits when using Mandarin for work and social communication. The new generation of migrant workers is a generation that has grown up in the context of the general promotion of Mandarin language use nationwide, and they are more inclined to use Mandarin in their work and daily life. The reasons for promoting the use of Mandarin for communication among new and old migrant workers are multiple, not only to gain social recognition and social integration, but also to meet the requirements of standardizing the use of Mandarin at work, breaking the communication barriers with urban people, and providing convenience for work and life, i.e., the use of Mandarin at work is motivated by survival needs. It is important to note that with the widespread promotion and popularization of Mandarin throughout the country, it is proposed in the Implementation Plan for the National Popularization of the Common Language and Script Project that by 2020, the national average penetration rate of Mandarin will reach over 80%. Mandarin is no longer a product unique to urban social life; it is a national lingua franca, a medium for people of different regions and nationalities in China to interact in production and life, and not a marker of a specific society, the city. Therefore hypothesis 1 is proposed.

H1: The language used at work has no significant effect on migrant workers’ willingness to settle in the city.

The language used by migrant workers in after-work communication is more a reflection of an emotional attachment and social group, which can reflect the social circle of social life as well as their living conditions. For example, the long-term background of working and living away from home makes them "homesick", and migrant workers who can communicate in the same dialect can obtain emotional comfort, while the hometown dialect is also a basis for migrant workers to find the same roots in a foreign place, and the same language habits can give individuals a higher sense of security. The language habits of migrant workers are not only based on social identity, but also on their factors, which are a reflection of both material and spiritual needs. Migrant workers who migrate to big cities to work make efforts to learn and use local dialects not only for work purposes but also to establish ties and social relationships with the city where they work. Local language, as a cultural factor, is also a feature in the accumulation of human capital of new and old migrant workers, which facilitates their advancement in the social development channel. Non-material cultural factors such as authority, status, and culture behind the language are also a kind of human capital. When they use the local dialect, they not only exchange information with the locals but also further enhance their social identity and connection with society. Therefore, from the perspective of language economics, the use of local dialects is a strategy for migrant workers to adapt to the city and to promote their identity as citizens. Based on the above analysis, Hypothesis 2 is proposed.

H2: The language used after work has a significant effect on migrant workers’ urban settlement.

In many research studies about migrant workers, it is shown that to better adapt to the working environment, migrant workers will show a strong willingness to learn the local language, through which they can not only complete their capacity building but also reflect their psychological changes in the process of integrating into the local society. At the same time, the increase in these language skills can not only bring improvements in work life but also change their situation [29]. For example, some scholars’ research in the PRD region found that Cantonese as a symbol of the local language contains the cultural connotation of the PRD behind it. Locally living residents are the main body of the urban discourse, and for migrant workers to be accepted by this urban discourse, they have to change their original dialect habits to gain a sense of identity [30]. In a survey of migrant workers in the Pearl River Delta region, Long Guolian also found that social interaction and willingness to migrate permanently had a more significant effect on the effect of migrant workers’ acquisition of the Cantonese dialect. The migrant population’s proficiency in the language of the inflowing region and frequent interactions with locals can positively influence their willingness to settle [31].

In this paper, we use the classification criteria of language economics to classify the types of language used by migrant workers when they go to work and after work, and study the factors that influence the differences in language use on migrant workers’ willingness to citizenship, focusing on the following issues: first, referring to Fu Yirong et al.’s study [28], we use the degree of migrant workers’ mastery of local dialects in their cities and the types of language they mainly use when they go to work and after work as the study investigated the impact of language use of migrant workers on their social integration in the city where they work and their willingness to settle in the city, using their mastery of the local dialect and the type of language they mainly use at work and after work as proxy variables for their language use in the city. Second, whether the degree of mastery of urban dialects and the language used on and off duty can be used as a proxy variable for language use differences depends on the formation mechanism of language use. This paper analyzes the intrinsic mechanism of different language use to answer why migrant workers who tend to use their home dialect after work develop a mentality of reluctance to integrate into the city, which is a test of "language economics". This is a validation of "language economics". Third, how does language use as an informal institution affect migrant workers’ willingness for citizenship? In addition to the direct effect of language use differences on willingness to citizenship, this paper further searches for intermediate variables between the two to explore the mechanisms by which language use differences affect willingness to citizenship.

In conclusion, Mandarin and urban dialects are both important influencing factors that affect migrant workers’ integration into cities and their acquisition of citizenship status, as well as important markers of their becoming citizens in the true sense of the word. Therefore, exploring the extent of migrant workers’ mastery of local dialects in their cities and the types of language they use at work and after work is important for studying migrant workers’ social integration in cities and their willingness to settle in cities. However, do the languages used by migrant workers at work and after work have different degrees of influence on their integration into the city and their willingness to settle there? Does migrant workers’ familiarity with the local dialect affect on their willingness to settle in the local area? At present, no scholars have explored this issue with empirical models for scientific argumentation. Based on the database of CLDS2016 National Labor Force Survey data from Sun Yat-sen University, this paper uses a multivariate ordered logistic model to explore the language used at work and the language and local dialect mastery after work and to answer the mechanism of its influence on migrant workers’ willingness to settle.

## 3. Construction of the empirical model

### 3.1 Data source

The data used in this paper come from the 2016 China Labor-force Dynamic Survey (CLDS) individual labor force addition questionnaire. The survey was conducted in 29 provinces, and municipalities directly under the central government and autonomous regions, and the data sample is nationally representative. In this paper, according to the needs of the questions, considering factors such as migration and work situation, we screen out the group of agricultural household registration laborers who are employed outside the farm and eliminate the questionnaire samples with important information missing, and finally have a sample of 2480.

### 3.2 Selection of indicators

The variables in this paper are defined as shown in Table 1. The dependent variable is the settlement intention of migrant workers in the city of employment, and the question of settlement intention in CLDS is "Will you settle in the local area in the future?" The answers are "very likely, relatively likely, uncertain, relatively unlikely, and very unlikely". The core independent variables include various aspects of language use by migrant workers in the city, such as the language they mainly use at work and the language they mainly use after work. First, the language is mainly used at work. Language is an important visual indicator of the environment in which migrant workers work, and the language they use at work can reflect their working environment. There are four options: Mandarin, the local dialect, the hometown dialect, and others. Second, the main language used after work. The language you mainly use after work is a visual representation of the living and social environment of migrant workers after work, and the CLDS2016 questionnaire asked respondents, "What is your main language after work?" The answers were Mandarin, the local dialect, hometown dialect, and other options. The control variables include personal characteristics of migrant workers such as age, education, and marital status, citizenship ability such as the ability to use Internet banking, purchase train tickets online, and withdraw money from ATMs, and human capital characteristics such as whether they have attended more than five days of technical training in the last six months and whether they have obtained professional and technical certificates. The degree of mastery of local dialects reflects the social integration and familiarity of migrant workers in the city of work. The more familiar with the dialect and culture of the city of work, the higher the migrant workers’ willingness to settle in the city of work. In this paper, we use the CLDS 2016 questionnaire to define the degree of mastery of local dialects by asking respondents to answer the question, "You know most of the local dialects, most of the local dialects, some of the local dialects, a little of the local dialects, and none of the local dialects". It was selected as a variable in the robustness analysis to further verify the effect of language usage habits on migrant workers’ willingness to settle in the city of work.

**Table 1:** Variable definition table.

Variables	Variable definition Mean	Mean	Std.dev
Willingness to settle	1 = very likely, 2 = more likely, 3 = uncertain, 4 = more unlikely, 5 = very unlikely	2.63	0.892
Language used at work	1 = Mandarin, 2 = Local dialect, 3 = Hometown dialect, 4 = Other	1.49	0.710
Language used at work	**1 = Mandarin, 2 = Local dialect, 3 = Old family dialect, 4 = Other**	2	0.834
Age	Continuous variable	43.61	12.567
Education	1 = never attended school, 2 = elementary school, private school 3 = junior high school, 4 = high school (including vocational high school and technical school), 5 = secondary school, college, 6 = bachelor’s degree and above	3.04	1.221
Marital status	1 = unmarried, 2 = married	1.88	0.324
Whether working in the county	1 = yes, 2 = no	1.77	0.419
Online train tickets	**0 = no problem at all, 1 = okay, 2 = not so good, 3 = not at all**	1.51	1.313
ATM withdrawal	0 = no problem at all, 1 = okay, 2 = not very good, 3 = not at all	0.91	1.245
Technical training	Whether attended more than 5 days of technical training in the last 6 months; 1 = yes, 2 = no	1.91	0.287
Technical certificate	**Whether to obtain professional technical certificate; 1 = yes, 2 = no**	1.88	0.324
Level of mastery of local dialect	1 = completely mastered, 2 = mostly mastered, 3 = partially mastered, 4 = a little mastered, 5 = not at all	2.33	1.589

### 3.3 Construction of the econometric model

The explanatory variable in this paper is the urban settlement intention of migrant workers, which includes five categories of ordered variables (1 = very likely ~ 5 = very unlikely) and is the dependent variable in discrete, ordinal form, so this paper uses a multivariate ordered logistic model for regression. The willingness to settle *Y*_*i*_ is assumed to be a linear function of *K* factor, and the value of this *K* factor is taken to be for the *i* individual migrant worker, as *X*_*ik*_(*k* = 1,2,3……,*k*) so the mathematical formula for willingness to settle can be expressed as:

$$Y_{i}=\sum_{k=1}^{k}b_{k}X_{ik}+e_{i}=Z_{i}+e_{i}$$ (1)

As shown in Eq 1, *β*_k_ are the *K* coefficients associated with the variable *X*_*ik*_(*k* = 1,2,3……,*k*), and $Zi=\sum_{k=1}^{k}\beta_{k}+X_{ik}$. If *β*_k_>0, for migrant workers, an increase in the value of the kth factor will lead to an increase in the willingness to settle, and vice versa it *β*_k_<0 will lead to a decrease in the willingness to settle.

## 4. Analysis of results

### 4.1 Descriptive analysis

In this study, the CLDS 2016 individual labor force questionnaire was used as the database, and the language use of migrant workers was used as the core variable to establish a multivariate ordered categorical logistic model of factors affecting migrant workers’ willingness to settle, and SPSS.22 was used to regress the data, and marginal coefficients were given based on when willingness to settle was very unlikely (i.e., willingness to settle took a value of 5). It is necessary to analyze the data descriptively before adopting the empirical model, to have an overall understanding of the settlement intention situation of migrant workers working outside. Reference is made to the definition of new-generation migrant workers and old-generation migrant workers in Zhang Fei’s study [32]. In general, among the old generation of migrant workers, the largest number of people said they were "very likely" to settle in the city, accounting for 25.35%; the percentage was higher than that of the new generation of migrant workers who said they were "very likely" to settle at 7.78%. Among the new generation of migrant workers, the highest percentage is among those who are "less likely" to settle in the city, accounting for 32.24%. Among the new generation of migrant workers, 23.17% of them are "more likely" to settle down, which is slightly higher than the proportion of 17.71% of the old generation. Generally speaking, the willingness to settle in cities of the new generation of migrant workers and the old generation of migrant workers is generally equal, and the difference between generations is small. Table 2 shows the specific distribution of urban settlement intention of new and old-generation migrant workers.

**Table 2:** Distribution of migrant workers’ willingness to settle.

Willingness to settle	New generation of migrant workers		Old generation of migrant workers	
	Frequency	Percentage	Frequency	Percentage
1 = Very likely	138	7.78%	179	25.35%
2 = More likely	411	23.17%	125	17.71%
3 = Uncertain	488	27.51%	173	24.50%
4 = Less likely	572	32.24%	107	15.16%
5 = Very unlikely	165	9.30%	122	17.28%
Total	1774	100%	706	100%

According to the survey, more than half of the rural migrant workers said they are unwilling to move their hukou to the city, and the rural hukou has the village collective contracted land, rural residential base, collective economic dividends, and special human culture in rural areas, which are the real factors that migrant workers are unwilling to give up their rural hukou. Secondly, the high housing price in cities is also an important reason that prevents migrant workers from settling in cities. Although some migrant workers have bought housing in cities earlier to solve the housing problem of settling in cities, most of them still find it difficult to settle in cities due to high housing prices. Migrant workers are limited by the pressure of higher prices in cities, and there is still a large gap between their income and urban housing prices.

### 4.2 Empirical results

In this paper, an ordered multi categorical logistic model is used to regress the language use of migrant workers on their intention to settle. Table 3 shows the results of the regressions. Model 1 and model 2 are the effects of language use at work and language use after work on migrant workers’ intention to settle, respectively, and model 3 is the regression result after considering both variables. The model regression is based on the last value in the definition of the variables. These two variables are based on "home dialect" as the base reference.

**Table 3:** Regression results of factors influencing migrant workers’ willingness to settle.

Variables	Model 1		Model 2		Model 3	
	Coefficient	Std.dev	Coefficient	Std.dev	Coefficient	Std.dev
Upper shift language	-0.475	0.291	-	-	-0.327	0.344
Upper shift language 2	-1.121	0.344	-	-	-1.005	0.759
Upper shift language 3	-	-	-	-	-	-
Off-shift language 1	-	-	1.281***	0.235	1.073***	0.569
Off-shift language 2	-	-	1.305***	0.268	1.115***	0.338
Off-shift language 3	-	-	-	-	-	-
Age	-0.012**	0.011	-0.015**	0.011	-0.05**	0.023
Degree 1	0.650	0.406	0.653	0.158	0.828	0.668
Degree 2	0.588	0.267	0.588	0.336	0.777	0.604
Degree 3	0.548	0.149	0.548	0.287	0.564	0.140
Degree 4	0.576	0.114	0.58	0.296	0.592	0.279
Degree 5	0.562	0.131	0.564	0.190	0.572	0.239
Degree 6	-	-	-	-	-	-
Marital status1	0.342*	0.113	0.343*	0.230	0.347*	0.148
Marital status2	-	-	-	-	-	-
Whether working in the county1	-1.385***	0.298	-1.293***	0.279	-1.010***	0.283
Whether working in the county2	-	-	-	-	-	-
Online train tickets0	1.298**	0.341	1.056**	0.346	0.877*	0.285
Online train tickets1	0.410	0.143	0.414	0.144	0.420	0.240
Online train tickets2	-0.982**	0.333	-0.613	0.336	-0.612**	0.338
Online train tickets3	-	-	-	-	-	-
ATM withdrawal 0	1.225***	0.354	0.950***	0.327	1.007***	0.356
ATM withdrawal 1	1.38*	0.433	0.910**	0.432	1.068*	0.436
ATM withdrawal 2	-1.308**	0.467	-0.944*	0.468	-1.097**	0.470
ATM withdrawal 3	-	-	-	-	-	-
Technical training1	0.389	0.283	0.394	0.152	0.397	0.215
Technical training2	-	-	-	-	-	-
Technical certificate 1	0.368*	0.211	0.366**	0.181	1.02*	0.367
Technical certificate 2	-	-	-	-	-	-

Note

***, **, * indicate significant at 1%, 5% and 10% statistical levels, respectively.

(1) Language used at work has no significant effect on migrant workers’ intention to settle in the city, and language used after work has a significant positive effect on migrant workers’ intention to settle in the city.

The language used at work variable in Model 1 does not have a significant effect on the coefficient of willingness to settle, i.e., the language mode mainly used at work does not have a significant effect on willingness to settle in the city, in line with the previous Hypothesis 1. The possible reason for this is that with the trend of widespread promotion of Mandarin throughout the country, migrant workers generally use Mandarin in the workplace. The use of Mandarin only facilitates work communication and is motivated by the need to survive and work, which does not include migrant workers’ emotional attachment. The use of Mandarin at work is not to gain a sense of urban integration and identity but is a requirement for work standardization. Therefore, the main language used at work has no significant effect on migrant workers’ intention to settle in the city of work.

The coefficient of the language variable used after work in model 2 is significant at the 1% level and has a significant positive effect on the intention to settle in the city, which verifies hypothesis 2. i.e., using the Hometown dialect as the base, the use of Mandarin and the local dialect after work both have a positive effect on the intention to settle in the city with coefficients of 1.281 and 1.305, respectively, and are significant at the 1% level. The language used by migrant workers after work is a manifestation of their social circle in urban life and is a manifestation of their emotional attachment. For example, the language used by migrant workers after work is their hometown dialect, which means that their social circle is mostly friends and relatives in their hometown, and they seek security from the same dialect. If the language used after work is Mandarin or the local dialect, it means that migrant workers in the city have a wider social circle during non-working hours and try hard to integrate into the life of the city. Language is also a kind of human capital accumulation, and Mandarin or local dialect, as part of the cultural composition of the city, is conducive to the promotion of migrant workers in their career development and integration into their social life. Therefore, the language mainly used after work is Mandarin or the local dialect has a significant positive effect on migrant workers’ willingness to settle in the city where they work.

(2) The effects of age, marital status, and in-county and out-of-county work situations on the intention to settle in the city.

From models 1, 2, and 3, it can be seen that age has a significant negative effect on urban settlement intention, and it is significant at the 5% statistical level. This is consistent with the findings of Zhenzhen Li et al. that migrant workers have certain intergenerational differences in their willingness to settle in cities and the older the migrant workers are, the more they have the sentiment of "returning to their roots" and want to go back to their hometowns to retire and live in rural areas [33]. Compared with married status, unmarried migrant workers have a stronger intention to settle in the city, which is statistically significant at the 10% level. Due to the difference in the ease of family settlement and individual settlement in the city, the factors influencing the urban settlement situation of married status are more complex, especially the education of children and the old age of the elderly in the family have notable pressure. With the development of Internet technology and the information age, the use of smart devices and the degree of adaptation to the smart age of migrant workers also have an important impact on urban settlement. In the econometric model migrant workers’ ability to purchase train tickets online on cell phones and withdraw money from ATMs has an impact on their willingness to settle in cities. That is, the urban settlement intention is higher for migrant workers who choose perfectly fine and okay for online train ticket purchase and bank ATM withdrawal ability. The urban settlement intention of migrant workers who work in the county is weaker compared to those who work outside the county, and it is significant at the 1% statistical level. Migrant workers who work in the county are closer to their hometowns and can return to their rural hometowns in a shorter period, so their intention to purchase commercial houses and settle in the city is weaker in the county towns. Migrant workers who work outside the county are more willing to buy commercial houses and settle in the city in addition to their homes in the rural areas because they spend more money on transportation and time on the road.

## 5. Robustness test

Through the analysis of the model estimation results above, the correlation between language use and urban settlement intention of the sample migrant workers is obtained, and then the robustness test will be conducted by the replacement indicator method, and the results are shown in Table 4. Model 4 in Table 4 represents the analysis of the relationship between local dialect familiarity and migrant workers’ willingness to settle. The robustness test is carried out from the perspective of variables, and the selection of indicators of language use variables of migrant workers is developed, and the local dialect familiarity variable is used as a substitute indicator of language use of migrant workers, which theoretically has the same attributes as the original indicator and can achieve a better test effect. In model 4, the local dialect level variable of migrant workers significantly and positively affects their urban settlement intention and is significant at the 1% level. That is, as migrant workers’ familiarity with the local dialect increases, their willingness to settle in the city becomes stronger. This variable also verifies hypothesis 2. When they learn the local dialect, they can not only further enhance their social identity, but also gain more information interchange.

**Table 4:** Regression results of factors influencing migrant workers’ willingness to settle.

Variables Model 4	Model 4	
	Coefficient	Std.dev
Local dialect 1	2.071***	1.291
Local dialect 2	2.013***	1.025
Local dialect 3	0.898*	0.390
Local dialect 4	0.399	0.149
Local dialect 5	-	-
Control variables	Controlled	Controlled

Note

***, **, and * indicate significant at the 1%, 5%, and 10% statistical levels, respectively

## 6. Discussion

This study aimed to investigate the influence of language use on the migrant workers’ willingness to Urban Settlement-based.Utilizing data of CLDS2016, we employed Logistics models to empirically examine the effects of language use on the migrant workers’ willingness to Urban Settlement-based. In addition, in order to further explore the influence of language use on migrant workers’ willingness to Urban Settlement-based, the use time is divided into working hours and after-work hours to explore the influence mechanism on migrant workers’ willingness to settle in cities respectively.

The findings revealed that the mastery of the urban dialect and the language used after work had a significant impact on the willingness of the migrant workers in the urban settlement, while the language used at work had no significant impact on the migrant workers’ willingness to settle in cities.

The contributions of this study are multifaceted and can be primarily observed in the following two dimensions.Firstly, the data survey was carried out in 29 provinces, municipalities and autonomous regions,it is nationally representative data samples.

Secondly, in previous studies, few scholars have conducted empirical analysis on the language use and settlement intention of migrant workers, and the literature only analyzes the correlation, without exploring the internal influence mechanism, nor further dividing the occasions and time of language use. Therefore, this study divides the occasions of language use of migrant workers into working hours and after-work hours. Then,this study explores the influence path of migrant workers’ language use on urban settlement willingness.

Although this study has made significant contributions to our understanding of the rela-tionship between Internet usage and the well-being of rural residents, there are still certain lim-itations that warrant further investigation.

Firstly, it is important to acknowledge that this study solely focused on data from the year 2016. Considering the dynamic nature of language use and its impact on migrant workers’ willingness to Urban Settlement-based, relying on data from a single year may provide a limited and static depiction of the phenomenon. Therefore, future research endeavors should aim to incorporate data spanning multiple years to provide a more comprehensive and longitudinal understanding of the effects of language use on the migrant workers’ willingness to Urban Settlement-based. By doing so, we can enhance the accuracy of the statistical results and gain a more realistic representation of the outcomes.

Secondly, although the data of 29 provinces were counted in this study, it did not take into account the possibility that language use of migrant workers in different regions has different effects on their willingness to settle in cities. This problem can become a direction of our team’s future research.By exploring regional differences, we can gain deeper insights into the nuanced dynamics of migrant workers’ language use and its impact on willingness to settle in cities.

In conclusion, this study divides language use time of migrant workers into working hours and after-work hours to explore the influence mechanism on migrant workers’ willingness to settle in cities. This study innovatively puts forward the influence factor of migrant workers’ language, which supplements the influence factor of migrant workers’ urban settlement willingness in previous literatures.Ultimately, this study is helpful to improve the willingness of migrant workers working in cities to settle down in the city, and has practical guiding significance.

## 7. Conclusions and implications

In this paper, using the data from the individual labor force questionnaire "CLDS2016", the language used at work, the language used after work, and the level of mastery of local dialects are considered as the core variables affecting the urban settlement intention of migrant workers, and a multivariate ordered logistic model is constructed to analyze the relationship between these three variables and urban settlement intention from the perspective of language economics. The study found that It was found that (1) language spoken at work had no significant effect on migrant workers’ willingness to settle in cities, language spoken after work had a significant positive effect on migrant workers’ willingness to settle in cities, and the level of the local dialect of migrant workers had a significant positive effect on their willingness to settle in cities. (2) Age has a significant negative effect on urban settlement intentions, and urban settlement intentions are stronger for unmarried migrant workers, and higher for those who choose perfectly fine and okay in their ability to purchase train tickets online and withdraw money from bank ATMs. Compared with those working outside the county, the urban settlement intention of migrant workers working in the county is weaker.

From the above analysis, it can be seen that to improve the urban settlement willingness of migrant workers working in cities, the following aspects can be considered.

(1) Improving the social integration of migrant workers and enhancing their sense of belonging to the city. In the after-work life, through grass-roots organizations such as residential communities, we can organize more activities that can enrich the spare time of migrant workers according to their life characteristics and habits, so that they can fully integrate into city life. By playing the role of public opinion in the city media, the important role of migrant workers in the city can be publicized to enhance the sense of belonging and pride of local migrant workers, and at the same time, residents in the city can better recognize migrant workers.

(2) To remove the obstacles and doubts of migrant workers and make the city livable and more suitable for settlement. To solve the problem of migrant workers’ difficulties in settling in cities, firstly, we should increase the scope of subsidized housing in cities and allocate part of the subsidized housing in cities to migrant migrant workers who contribute more to the city to buy or rent; secondly, for migrant migrant workers who have formed families, we should appropriately relax the enrollment policy in the education of their children, to solve the education problem of their children and increase their willingness to settle in cities.
